# Leukemic penile ulcer as the presenting symptom of chronic lymphatic leukemia

**DOI:** 10.1093/jscr/rjaf1101

**Published:** 2026-01-27

**Authors:** Harsahib Singh Sandhu, Suresh Chandra, Smiley Joshi

**Affiliations:** MBBS Intern, Kasturba Medical College, Manipal, Manipal Academy of Higher Education, Tiger Circle Road, Madhav Nagar, Manipal, Karnataka, 576104, India; Director Histopathology, Medgenome Labs Ltd, Hosur Road, Bengaluru, Karnataka, 560100, India; Consultant Medical Oncologist, Sandhu Cancer Centre, Udham Singh Nagar, Civil Lines, Ludhiana, Punjab, 141001, India

**Keywords:** penile ulcer, penile small lymphocytic lymphoma, Bruton tyrosine kinase inhibitors, acalabrutinib, extranodal small lymphocytic lymphoma

## Abstract

Chronic lymphatic leukemia (CLL) is the most common type of leukemia in the West, with an incidence of 4.2 per 100 000 population. Penile prepuce involvement at presentation, as the first manifestation of CLL, is extremely rare. We have seen a 69-year-old male patient who presented with a non-healing ulcer on the foreskin of the penis for 6 weeks. Biopsy revealed the diagnosis of small lymphocytic lymphoma. His hematological work-up uncovered an underlying CLL. He was treated with acalabrutinib, a Bruton tyrosine kinase inhibitor, resulting in complete remission after 7 weeks. In the vast majority of patients with CLL, the disease primarily involves bone marrow, peripheral blood, lymph nodes, and spleen; extramedullary and extranodal involvement is rarely seen. This case highlights the importance of keeping leukemic involvement into consideration in patients with penile lesions, as it can be the first manifestation of underlying CLL.

## Introduction

Leukemic penile ulcer in chronic lymphatic leukemia (CLL) as the first manifestation of disease is extremely rare [1], with only a few cases reported in the medical literature [2], as the disease primarily involves bone marrow, peripheral blood, lymph nodes, and spleen [3]. In this report, we describe a patient who presented with an ulcerative lesion on the prepuce, which turned out to be small lymphocytic lymphoma (SLL) on histopathology, and the hematological workup revealed the diagnosis of underlying CLL.

## Case report

**Figure 1:**
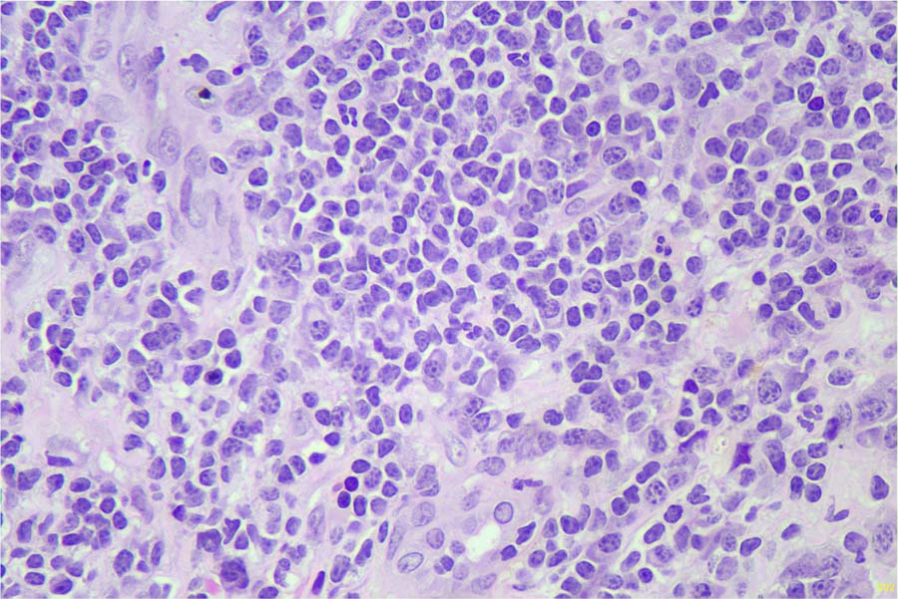
Hematoxylin and eosin (H&E) stain shows small lymphoid cells in sheets (400×).

A 69-year-old male presented with a painful, non-healing ulcer on the foreskin of the penis for 6 weeks. Examination revealed a tender, ulcerative, erythematous lesion on the left side of the inner prepuce measuring 2 × 3 cm. A biopsy from the penile ulcer was done, and the histopathology revealed a dense lymphoid infiltrate with small, monomorphic, round cells resembling mature lymphocytes (Fig. 1). On immunohistochemistry (IHC), the lymphocytes were positive for CD5, CD20, and CD23, while CD10 and Cyclin D1 were negative, consistent with the diagnosis of SLL of the prepuce. The Ki-67 score was 20%. His hemogram revealed a hemoglobin of 7.0 g/dl (reference range 13–17 g/dl), white blood cell count of 102.8 × 10^3^/ul (4–11 × 10^3^/ul), and platelet count of 41.0 × 10^3^/ul (150–400 × 10^3^/ul). The peripheral smear showed lymphocytosis with 91% mature-looking lymphocytes along with smudge cells. Immunophenotyping by flow cytometry on peripheral blood using the chronic lymphoproliferative disorder (CLPD) panel revealed a monoclonal B-cell population of 92% lymphocytes with moderate expression of CD5, CD19, CD23, CD43, and CD200, dim expression of CD20, CD22, and CD38, and bright expression of CD45. These cells were lambda light chain restricted and negative for CD10, CD11c, CD103, and FMC7. Hence, the final diagnosis of CLL with penile involvement was made. Coomb’s test, both direct and indirect, was negative. Serum LDH was raised, 245.50 u/l (135–225 u/l). Bone marrow aspiration and biopsy showed a hypercellular marrow with diffuse and interstitial patterns of involvement of 90% of the marrow space by small round lymphoid cells, with these cells constituting 94% of the total cell population. The chromosomal analysis showed a normal karyotype (46, XY). Fluorescent *in situ* hybridization (FISH) for the CLL panel was negative for Deletion 6q, Deletion 11q, Trisomy 12, Deletion 13q, Deletion 17p, and IGH gene rearrangement. IGHV gene mutation analysis by Sanger sequencing showed an unmutated IGHV gene. The next-generation sequencing (NGS) leukemia panel showed an SF3B1 p.Lys700Glu missense gene mutation with a variant allele frequency (VAF) of 47.44%. The computed tomography (CT) scan showed bilateral cervical, axillary, inguinal, and femoral, as well as abdominal and pelvic lymphadenopathy. Hepatomegaly (19 cm) and splenomegaly (19.5 cm) were also present. Based on the workup, a diagnosis of high-risk modified Rai stage IV CLL with prepuce involvement was made.

He was treated with acalabrutinib, a Bruton tyrosine kinase inhibitor, 100 mg twice daily. After 7 weeks of treatment, there was complete remission of the penile lesion, and he became symptom-free. He continues to be in complete remission at a follow-up of 1 year and 9 months.

## Discussion

In the vast majority of patients with CLL, the disease primarily involves bone marrow, peripheral blood, lymph nodes, and spleen; extramedullary and extranodal involvement is very rare [4]. Penile involvement is exceedingly rare, occurring in cases with a prolonged history of CLL years into the course of the disease [5], while our case presented with prepuce involvement by SLL as the first manifestation of disease, and the diagnosis of CLL was made during the routine hematological workup. Penile involvement by second primary cancers like squamous cell carcinoma of the penis is much more common in long-standing cases of CLL than primary penile involvement by CLL [4]. In patients with a long history of CLL, the penis can also get involved by metastasis from second primary cancers of the prostate, bladder, colon, and kidneys [2, 5].

Morphologically, the penile lesions in CLL can be very variable, ranging from macules, papules, plaques, nodules, diffuse penile swelling, non-healing ulcers (as in our case), or even malignant priapism [3], and these lesions can involve the glans, prepuce, coronal sulcus, or shaft of the penis [1]. Thus, the penile lesions are nonspecific, and the distinction from benign lesions can be challenging. To make an early diagnosis, a high index of suspicion is required. Timely penile biopsy, along with IHC, goes a long way in making the timely diagnosis. The surgeon and pathologist need to keep CLL as a possibility in a non-healing penile lesion to prevent delay in diagnosis, as the infiltrate in CLL may appear mature and can be mistaken for a nonspecific chronic inflammatory infiltrate. Temptation to start topical steroid creams should be avoided before biopsy, as these can potentially interfere with the interpretation of histopathological features.

The penile involvement in CLL is considered a sign of a poor prognosis, increased risk of Richter’s transformation, and a shorter survival time [4, 6]. Moreover, in our case, there were many other adverse prognostic features, including an unmutated IGHV gene [7], the presence of the SF3B1 p.Lys700Glu missense gene mutation [8], diffuse pattern of bone marrow infiltration [9], expression of CD38 [10], raised serum LDH [11], and high-risk modified Rai stage IV disease [12]. However, despite the presence of so many poor prognostic features, our patient responded very well to acalabrutinib, a Bruton tyrosine kinase (BTK) inhibitor, and continues to be in complete remission at 1 year, 9 months of follow-up, demonstrating the responsiveness of the penile lesion even with adverse prognostic features. With the advent of targeted therapies, radical surgery like a penectomy is no longer required in such cases. The patients who progress on single-agent acalabrutinib, a combination of acalabrutinib and venetoclax, a B-cell lymphoma-2 (BCL-2) inhibitor, is effective and can result in complete remission [2].

This case highlights the importance of keeping leukemic involvement into consideration in patients with penile lesions, as it can be the first manifestation of underlying CLL.

## Data Availability

The data that support the findings of this study are available from the corresponding author upon reasonable request.
